# *Artemisia santonicum* L. and *Artemisia lerchiana* Web. Essential Oils and Exudates as Sources of Compounds with Pesticidal Action

**DOI:** 10.3390/plants12193491

**Published:** 2023-10-06

**Authors:** Milena Nikolova, Aneta Lyubenova, Elina Yankova-Tsvetkova, Borislav Georgiev, Strahil Berkov, Ina Aneva, Antoaneta Trendafilova

**Affiliations:** 1 Institute of Biodiversity and Ecosystem Research, Bulgarian Academy of Sciences, 1113 Sofia, Bulgaria; e_jankova@abv.bg (E.Y.-T.); bobogeorgiev5@gmail.com (B.G.); berkov_str@yahoo.com (S.B.); ina.aneva@abv.bg (I.A.); 2 Department of Agrobiotechnology, AgroBioInstitute, Agricultural Academy, 1164 Sofia, Bulgaria; anetalyubenova@abi.bg; 3 Institute of Organic Chemistry with Centre of Phytochemistry, Bulgarian Academy of Sciences, 1113 Sofia, Bulgaria; antoaneta.trendafilova@orgchm.bas.bg

**Keywords:** acetylcholinesterase, biopesticides, seed germination, phytopathogenic fungi, *Botrytis cinerea*

## Abstract

The application of natural products for pest control is important in modern farming. In the present study, *Artemisia santonicum* L. and *Artemisia lerchiana* Weber essential oil and exudate profiles were determined, and their potential as inhibitors of seed germination, acetylcholinesterase, and phytopathogenic mycelium growth were evaluated. Essential oils (EO) were obtained via hydrodistillation and exudates (AE) by washing aerial parts of the species with acetone. EO and AE’s composition was identified using GC/MS. Eucalyptol (1,8-cineole) and camphor were found to be the main components of *A. lerchiana* EO, while β-pinene, *trans*-pinocarveol, α-pinene, α-terpineol, and spathulenol were established as major compounds of *A. santonicum* EO. Strong inhibition on *Lolium perenne* seed germination was found at 2 µL/mL and 5 mg/mL using aqueous solutions of EO and AE, respectively. An inhibitory effect on acetylcholinesterase was established, with an IC_50_ value of 64.42 and 14.60 μg/mL for EO and 0.961, >1 mg/mL for the AE of *A. lerchiana* and *A. santonicum*, respectively. The low inhibition on the mycelium growth of studied phytopathogenic fungi was established by applying 2 µL of EO and 15 µL of 100 mg/mL of AE, with the exception of *A. lerchiana* AE against *Botrytis cinerea*. These results show that the studied EO and AE exhibited strong phytotoxic and AChE inhibitory activities, providing new data for these species.

## 1. Introduction

Environmental pollution caused by synthetic pesticides, as well as pest resistance to them, requires a continuous search for new natural sources of biocidal activity [1,2,3,4,5]. In recent decades, the interest in studying essential oils and plant extracts as new natural remedies for pest control has increased [4,6,7,8,9]. A lot of research points out essential oils as promising agents for the control of crop pests—weeds, insects, and phytopathogens [10,11,12,13,14]. As exudates are composed of substances located on the surface of plant tissues, they have chemoecological importance as protectors against pests, diseases, and UV radiation. Available data point out that exudates possess plant-growing inhibitory, antimicrobial, antiplasmodial, antifeedant, and insecticidal activities [15,16,17,18,19,20].

The Asteraceae family has been found to be a good source of compounds with biocidal activity [21,22,23,24,25]. Flowers from *Tanacetum cinerariifolium* (Trevir.) Sch. Bip. (syn. *Chrysanthemum cinerariaefolium* (Trevir.) Vis.; *Pyrethrum cinerariifolium* Trevir.) are the best-known example of commercially available plant insecticide [26]. Phytotoxic, antifungal, and insecticidal activities have been reported for essential oils and extracts from several *Artemisia* species [24,25,27,28]. Based on these facts, we directed our interest to study the pesticide properties of two closely related species with limited distribution in Bulgaria—*Artemisia santonicum* L. and *Artemisia lerchiana* Weber. The essential oils of these species are of various origins and have been studied before [29,30,31,32,33], while their biological activities have been insufficiently studied. Insecticidal properties against *Sitophilus granarius* adults and antifungal activity against *Aspergillus* sp., *Penicillium* sp., and *Trichoderma viride*, as well as antibacterial activity, have been reported for *Artemisia santonicum* EO [33,34,35]. The antifungal activity of *Artemisia lerchiana* EO on *Sclerotinia sclerotiorum* has been reported [36]. Data concerning the composition of exudates on both species have been reported in our previous work [37]. Still, regarding biological action, there is only information on their antibacterial activity [38].

The aim of the present study is to determine *A. santonicum* and *A. lerchiana* essential oil and exudate profiles and to evaluate their potential as inhibitors of seed germination, acetylcholinesterase, and the mycelium growth of phytopathogenic fungi. The inhibition of acetylcholinesterase exists at the base of the mechanism of action of highly toxic organophosphate insecticides [39,40]. We consider this effect as a measure of the insecticidal activity of the investigated fractions and an important indicator of their effectiveness.

## 2. Results

### 2.1. Phytochemical Analysis

The GC/MS analysis of essential oils obtained from the aerial parts of *Artemisia santonicum* and *A. lerchiana* led to the identification of 83 compounds accounting for 98.1% and 97.1% of the total oil, respectively (Table 1). Out of them, 79 compounds were unambiguously identified, and 4 were determined as 3 sesquiterpene alcohols and a sesquiterpene lactone based on their mass-spectral fragmentation. Oxygenated monoterpenes (62.8%) were the predominant class of compounds in *A. lerchiana* essential oil, followed by oxygenated sesquiterpenes (24.4%) and monoterpene hydrocarbons (8.2%). *A. santonicum* essential oil contained almost equal amounts of monoterpene hydrocarbons (34.0%) and oxygenated monoterpenes (35.9%), followed by O-containing sesquiterpenes (19.5%). Both essential oils were poor in sesquiterpene hydrocarbons. The essential oils differed significantly in the content of individual components too. Thus, eucalyptol (22.1%) and camphor (14.0%) were the main components of *A. lerchiana* essential oil, while β-pinene (15.2%), *trans*-pinocarveol (9.6%), α-pinene (9.3%), α-terpineol (9.2%), and spathulenol (8.5%) were the major compounds of *A. santonicum* essential oil.

The identified compounds of exudates using GC/MS are presented in Table 2. Primary and secondary metabolites were established. Monoterpenes, phenolic acids, sugar alcohols, triterpenes, and flavonoid aglycones were determined as the main bioactive compounds. Acetone exudate of *A. lerchiana* was found to be rich in monoterpenes—eucalyptol (6.45%), camphor (4.58%), borneol (3.69%), carvacrol (1.86%) and ascaridole (1.49%). 10-Undecenoic acid (11.45%) was identified as a fatty acid in large amounts. The exudate profile of *A. santonicum* organic acids—malic (5.72%) and fumaric (4.25%)—were found to be the most abundant. Pinitol (3.61%), erythronic acid (2.45%), hydroquinone (1.90%), capillene (1.32%), cinnamic acid (0.95%), and chlorogenic acid (0.68%) were also identified as the main bioactive compounds in the exudate.

### 2.2. Inhibition of Seed Germination

**Figure 1:**
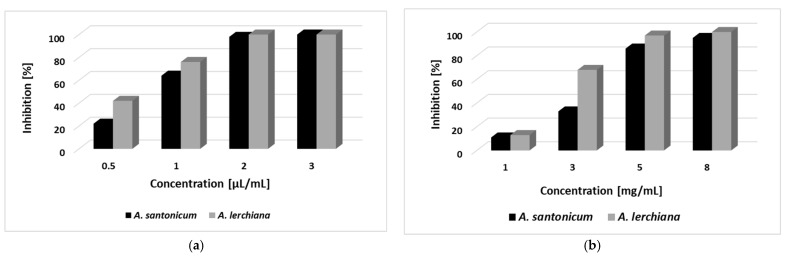
Inhibition on *L. perenne* seed germination using EO (**a**) and AE (**b**) of the studied species.

The inhibitory activity of aqueous solutions of *A. lerchiana* and *A. santonicum* EO and AE on the germination of *Lolium perenne* seeds was assessed. The results are presented in Figure 1. The strong inhibition of seed germination was achieved by applying aqueous solutions of essential oils at a 2 µL/mL concentration. *Artemisia lerchiana* EO showed slightly higher phytotoxicity compared to that of *A. santonicum.* Applying exudates at 5 mg/mL determined the strong inhibition of seed germination. Again, *A. lerchiana* AE exhibited stronger phytotoxicity.

### 2.3. Inhibition of Acetylcholinesterase (AChE)

The essential oils and exudates of the studied species were evaluated for their AchE inhibitory activity. The results are shown in Table 3. None of the tested samples were proven to be a stronger AchE inhibitor than galanthamine. Both Eos exhibited potent enzyme inhibition, but *A. santonicum* EO showed a lower IC_50_ value, indicating a higher AchE inhibitory activity than *A. lerchiana*. The exudates of both species showed similar activity, with IC_50_ values much higher than Eos. Therefore, Aes can be considered weak AchE inhibitors.

### 2.4. Inhibition of Phytopathogenic Mycelium Growth

**Figure 2:**
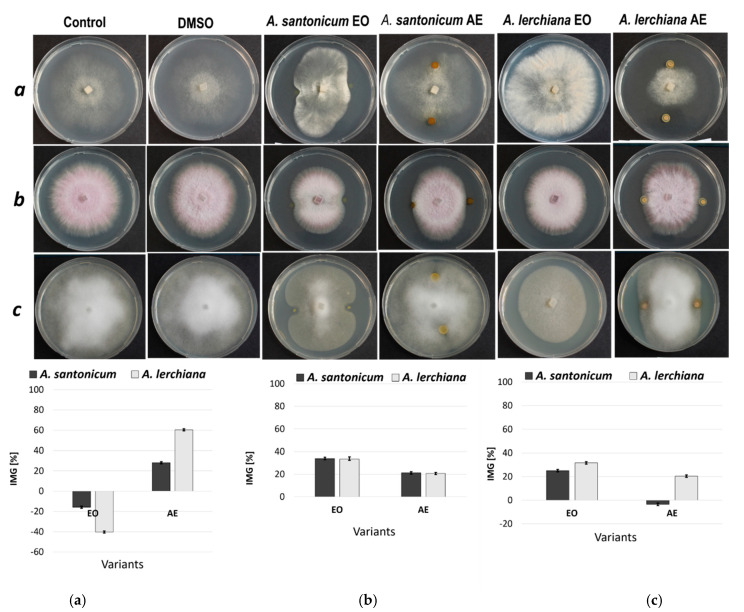
Antifungal activity of *A. santonicum* and *A. lerchiana* essential oils (EO) and acetone exudates (AE) in vitro, after 6 days at 25 °C (***a***–***c***), against *Botrytis cinerea* (**a**), *Fusarium oxysporum* (**b**) and *Phytophthora cryptogea* (**c**). Error bars represent the standard error of the mean (*n* = 4). %IMG—Inhibition of mycelial growth.

The inhibitory effect of essential oils and acetone exudates of *A. lerchiana* and *A. santonicum* on the mycelium growth of *Phytophthora cryptogea*, *Botrytis cinerea*, and *Fusarium oxysporum* was assessed. The results are presented in Figure 2. The slightly inhibiting effect on *F. oxysporum* and *P. cryptogea* mycelium growth (34% and 25% IMG, respectively) was established for *A. santonicum* EO, while an opposite effect toward *B. cinerea* was found. These fungi formed a larger and denser colony than the control (Figure 2). The essential oil of the second plant species, *A. lerchiana*, had a stronger inhibitory activity on *F. oxysporum* (33% IMG) and *P. cryptogea* (31% IMG) mycelium growth than *A. santonicum* EO. The stimulating effect on *B. cinerea* growth was found once again with *A. lerchiana* EO, even more pronounced.

The acetone exudate of *A. Lerchiana* had the strongest inhibitory effect on the growth of colonies of *B. cinerea* (60% IMG) and was weaker on the growth of *F. oxyisporum* and *P. cryptogea* (20% IMG for both).

The *A. santonicum* AE exhibited a minor inhibitory effect on *F. oxysporum* and B*. cinerea* growth (21% and 28% IMG, respectively), whereas in the *P. cryptogea* variant, the stimulating effect on mycelium growth was observed again, but much weaker compared to the Eos against *B. cinerea*.

The studied *A. santonicum* and *A. lerchiana* EO and AE showed low antifungal activity against the tested phytopathogenic isolates at the applied concentrations of 2 µL and 15 µL from 100 mg/mL for EO and AE, respectively.

## 3. Discussion

### 3.1. Phytochemical Analysis

The established composition of the essential oil *A. lerchiana* was in accordance with previously reported data for Bulgarian, Romanian, and Russian populations of this species [29,30,31]. The literature survey on the essential oil profile of *A. santonicum* showed significant variability in major components depending on the origin. Thus, eucalyptol (1,8-cineole), chrysanthenone, and *cis*-thujone were reported as the main components in the oil from a Serbian population [41]. At the same time, camphor was found to be the principal compound in the plant material from Turkish populations [32,35,42]. The main components of the *A. santonicum* profile established in the present study (β-pinene, *trans*-pinocarveol, α-pinene, α-terpineol, and spathulenol) were different from those reported for other populations [32,35,41,42]. This great variability in the essential oil profile of this species could be related to the fact that this species is considered a very variable taxon from the *A. maritima* group [43].

Data on the exudate composition of the studied species have been previously reported by our research group only [37]. In the present study, information on the composition of the exudates was added with data from several newly identified bioactive compounds such as carvacrol, hydroquinone, capillene, pinitol in *A. santonicum* exudate and eucalyptol, camphor, carvacrol, borneol and 10-undecenoic acid in *A. lerchiana* exudate. Based on TLC analysis in our previous report [37], the exudates of both species are rich in flavonoid aglycones. Luteolin, apigenin, 6-hydroxyluteolin 6-methyl ether, 6-hydroxyluteolin 6,3′-dimethyl ether, scutelarein 6-methyl ether, and scutelarein 6,4′-dimethyl ether have been reported as common flavonoid aglycones for both species. Quercetin, quercetagetin 6-methyl ether, and apigenin 4-methyl ether have been found in *A. santhonicum* exudate, while quercetagetin 3,6,4′-trimethyl ether has been found in *A. lerchiana* exudate [37]. GC/MS analysis with the derivatization method used in the present study did not allow the identification of a large part of the flavonoid aglycones.

### 3.2. Inhibition on Seed Germination

The observed strong inhibition of seed germination with aqueous solutions of studied essential oils at 2 µL/mL indicates significant inhibitory activity. This value is comparable to the values reported for essential oils considered as promising for application as bioherbicides, such as *Satureja hortensis*, *S. montana*, *Mentha piperita*, *Peumus boldus*, *Origanum vulgare* ssp. *hirtum*, *Artemisia annua*, *Artemisia scoparia* and *Cymbopogon citratus.* The total inhibitory activity in the 4–5 µL/mL range has been determined as strong [24,28,44,45,46]. *Artemisia lerchiana* EO is slightly more active compared to *A. santonicum* EO. Both main compounds (1,8-cineole for *A. lerchiana* EO and β-pinene for *A. santonicum* EO) are considered to possess strong phytotoxic properties [24,47,48]. Fagodia et al. [49] reported that 1,8-cineole shows a greater inhibitory activity on seed germination than β-pinene. This is a probable reason for the difference observed in the inhibitory activity of studied essential oils.

More than 90% inhibition of seed germination was achieved by the application of aqueous solutions of AE at 5 mg/mL. This result shows the strong inhibitory activity of the studied exudates because such inhibition has been reported by applying plant extracts at much higher concentrations [7]. Over 90% inhibition after applying the *Cardus cardunculus* (Asteraceae) crude extract has been observed at a concentration of 10 g L^−1^ [50]. The herbicide potential of *A. lerchiana* and *A. santonicum* EO and AE were evaluated for the first time in the present study.

### 3.3. Inhibition of Acetylcholinesterase (AChE)

Obtained data for the AChE inhibitory activity of studied oils has shown high potential comparable to that of *A. dracunculus* essential oil [51]. The stronger AChE inhibition caused by *A. santonicum* EO compared to *A. lerchiana* EO can be explained by the higher β-pinene content. It has been reported that monoterpene β-pinene displays better AChE activity than 1,8-cineole, which is the main component of *A. lerchiana* EO [52,53].

*A. santonicum* EO has been previously investigated for inhibitory activity on acetyl- and butyrylcholinesterase, but the IC_50_ value has not been determined [42]. Thus, here we report for the first time the IC_50_ values for acetylcholinesterase inhibitory activity of *A. lerchiana* and *A. santonicum* EO and AE.

### 3.4. Inhibition of Phytopathogenic Mycelium Growth

The essential oils of both species showed minor inhibitory activity on the mycelium growth of the tested phytopathogens at applied doses: 2 μL and 15 μL from 100 mg/mL stock solution for EO and AE, respectively. Kordali et al. [54] reported the inhibitory activity of *A. santonicum* EO against fungi, but the EO applied was 10 μL and 40 μL per Petri dish. The concentration selected for screening in the present study was based on data in the literature that determined the presence of antifungal activity in the oil at a concentration range (0.05–5 μg/mL) as strong [55,56]. It has been reported that essential oils from *Cinnamomum zeylanicum*, *Cananga odorata*, *Ocimum basilicum*, *Cymbopogon citratus*, *Boswellia thurifera*, *Majorana hortensis* at a concentration of 1 μL/mL inhibit the growth of *Aspergillus niger*, which is a common spoilage fungus [55]. The essential oil of *Origanum vulgare* ssp. *hirtum* at concentrations of 0.2–0.8 μL/mL was reported to inhibit the growth of *Fusarium solani*, *F. oxysporum*, *Alternaria solani*, *A. Alternata*, and *Botrytis cinerea* [57].

Several recent studies have investigated the antimicrobial properties of different *Artemisia* spp. Trifan and colleagues reported that *Artemisia* root extracts exhibit strong anti-Mycobacterium effects. Chloroform and methanol extracts obtained from the roots and aerial parts of five *Artemisia* species displayed good antibacterial effects against Mycobacterium tuberculosis H37Ra, with MIC values of 64–256 mg/L [58]. In another study, the antibacterial effects of leaf and stem ethanolic extracts of *Artemisia absinthium* L. and *Artemisia annua* L. on clinically important pathogenic bacteria were assessed. Artemisia extracts exhibited potent antibacterial activity against *E. coli*, *S. aureus*, *L. monocytogenes*, *S. enteritidis*, and *Klebsiella* sp. [59]. The essential oil of *Artemisia negrei* L., a species that is widespread in Morocco, Africa, also demonstrated potent antibacterial activity toward several multidrug-resistant bacteria. In the same study, antifungal activity with a percentage inhibition of 32%, 33%, and 33% was demonstrated against the fungal pathogens *F. oxysporum*, *A. niger*, and *C. albicans* at a dosage of 10 μL of essential oils on a Petri dish, which is at least twice that demonstrated in the current study [60].

The observed stimulating effect on the phytopathogenic mycelium growth is not unusual. Slavov et al. [61] previously reported that the acetone exudate and aqueous-methanolic extract of *Tagetes patula*, as well as the aqueous-methanolic extract of *Tanacetum vulgare*, enlarge the size of the mycelium colonies of *Phytophthora cambivora* when applied in vitro. This reaction could be attributed to the presence of sugars in the extracts and exudates, which were used in the cited study.

## 4. Materials and Methods

### 4.1. Plant Material

Aerial parts of the studied species were collected from the Bulgarian Black Sea coast in the summer of 2020 in the flowering phenological stage. *A. lerchiana* was collected from Byalata laguna locality, close to Balchik (43°24′24.92″ N/28°14′31.21″ E, 31 m a.s.l.). *A. santonicum* was collected from a locality close to Primorsko (42°14′42.49″ N/27°45′22.82″ E, 13 m a.s.l.). Plant species were identified by Dr. Ina Aneva. Voucher specimens—CO 1435 (*A. santonicum*) and CO 1436 (*A. lerchiana*)—were deposited at the Herbarium, Institute of Biodiversity and Ecosystem Research (SOM), Bulgarian Academy of Sciences, Bulgaria. The collected plant material was dried at room temperature (around 25*֩*C) without direct sunlight.

### 4.2. Extraction Procedures

The essential oils were extracted from dried aerial parts of the studied species on a Clevenger apparatus via water distillation for 2 h. The extraction process was repeated several times to obtain the necessary amount of essential oil for the experiments. The oils were dried over anhydrous sodium sulfate and stored at −4 °C in a sealed vial until required. Exudates were obtained according to the method introduced by Prof. Eckhart Wollenweber to study exudate flavonoids but without removing substances with a terpenoid structure in the present study [62,63]. Dry rather than ground aerial parts of both species were dipped into acetone for 5 min to dissolve the material accumulated on the surface of plant tissues and secretory structures. The obtained exudates were filtered, and the acetone was removed using a rotary vacuum evaporator.

### 4.3. Derivatization of the Exudates

In total, 100 µL of pyridine and 100 µL of *N*,*O*-bis-(trimethylsilyl)trifluoroacetamide (BSTFA) were added to the dried samples of AE, and they were heated at 70 °C for 2 h. After cooling, 300 μL of chloroform was added, and the samples were analysed using GC/MS.

### 4.4. GC/MS Analysis

GC/MS spectra were recorded on a Thermo Scientific Focus GC coupled with a Thermo Scientific DSQ mass detector operating in the EI mode at 70 eV (Thermo Fisher Scientific, Waltham, MA, USA). The ADB-5MS column (30 m × 0.25 mm × 0.25 μm) was used. The chromatographic conditions were as follows: helium was the carrier gas at a flow rate of 1 mL/min; the injection volume was 1 μL; and the split ratio was 1:50. Column temperature was 60 °C for 10 min, programmed at the rate of 3 °C/min to 200 °C, and finally, held isothermally for 10 min. The injection port was set at 220 °C. The significant quadrupole MS operating parameters were as follows: interface temperature of 240 °C; electron impact ionization at 70 eV with a scan mass range of 40 to 400 *m*/*z* at a sampling rate of 1.0 scan/s [64]. The temperature program for the analysis of AE samples was: 100–180 °C at 15 °C × min^−1^, 180–300 °C at 5 °C × min^−1^ and 10 min hold at 300 °C. The injector temperature was 250 °C. The flow rate of carrier gas (Helium) was 0.8 mL × min^−1^. The split ratio was 1:10. In total, 1 mL of the solution was injected [65].

Relative percentage amounts of the compounds were calculated from TIC. Relative retention indices (RRI) of the compounds were calculated using retention times of C_8_–C_25_ *n*-alkanes under the same chromatographic conditions. The individual components were identified by their MS and RRI values, referring to known compounds from the literature [66,67] and also by comparison with those of the NIST 14 Library and homemade MS databases.

### 4.5. Preparation of Fractions (EO and AE) before Bioassays

Table 4 summarizes the solvents and concentrations of EO and AE used in the different bioassays. Details of fraction preparation are included in the descriptions of the individual bioassay.

### 4.6. Inhibition on Seed Germination

The seed germination experiment was performed in laboratory conditions. A hundred seeds of *Lolium perenne* (a common crop weed) were placed per Petri dish on filter papers moistened with the tested solutions. Aqueous solutions of the essential oils at a concentration of 0.5, 1, 2, and 3.0 μL/mL were prepared using 0.1% Tween 40 (Sigma) as an emulsifier. The solutions were processed on Vortex. The exudates were dissolved in a water–acetone mixture (99.5:0.5) and were assayed at concentrations of 1, 3, 5, and 8 mg/mL. The two control solutions consisted only of 0.1% Tween 40 or a water–acetone mixture for EO and AE, respectively. The samples were incubated at room temperature for seven days. At the end of the week, the rate of germination inhibition [%] was calculated, as described by Atak et al. [68]:

GI = [(GC − TG)/GC] × 100,

where GI is the rate of germination inhibition (%); GC is the germination rate of seeds treated with control solutions; and TG is the germination rate of seeds treated with an EO or AE solution. Experiments were performed in three independent replicates.

### 4.7. Inhibition of Acetylcholinesterase (AChE)

Acetylcholinesterase inhibitory activity was determined using Ellman’s colorimetric method, as modified by López et al. [69]. First, all samples were dissolved in 10% MeOH at a concentration of 10 mg/mL. Next, they were serially diluted 10-fold using a phosphate buffer (PBS) (8 mM K_2_HPO_4_, 2.3 mM NaH_2_PO_4_, 0.15 M NaCl, pH 7.5) to provide the concentration range needed. AE and EO solutions with seven different concentrations from 0.001 to 1000 µg/mL were tested. Acetylthiocholine iodide (ATCI) in a solution with 5,5′-dithiobis(2-nitrobenzoic acid) (DTNB) was used as a substrate for AChE from *Electrophorus electricus* (Sigma-Aldrich, Taufkirchen, Germany) (0.04 M Na_2_HPO_4_, 0.2 mM DTNB, 0.24 mM ATCI, pH 7.5).

Then, 50 µL of AChE (0.25 U/mL) dissolved in PBS and 50 µL of the tested sample solution were added to the wells. The incubation of the plates was performed at room temperature for 30 min. Then, 100 µL of the substrate solution was added to start the enzymatic reaction. The absorbances were read in a microplate reader (BIOBASE, ELISA-EL10A, Jinan, China) at 405 nm after 3 min. Enzyme activity was calculated as an inhibition percentage compared to an assay including a buffer instead of an inhibitor. Galanthamine was used as a positive control. All data were analyzed with the software package Prism version 3.00 (Graph Pad Inc., San Diego, CA, USA). The IC_50_ values (half maximal inhibitory concentration) for AEs and EOs were measured in triplicate, and the results are presented as means ± SD.

### 4.8. Inhibition of Phytopathogenic Mycelium Growth

We investigated the impact of *A. santonicum* and *A. lerchiana* essentials oils and acetone exudates on the mycelium growth of three economically important plant pathogens—*Botrytis cinerea, Fusarium oxisporum* and *Phytophthora cryptogea*—which were obtained from agricultural ecosystems in Bulgaria and deposited in the fungal collection of AgroBioInstitute, Agricultural Academy. The plant pathogens were identified using classical methods and molecular techniques based on the sequencing of the ITS region of rRNA genes [70]. *P. cryptogea* was obtained by baiting, according to Jung and Blaschle [71], of the rhizosphere soil of *Rubus idaeus*. *F. oxysporum* and *B. cinerea* were previously isolated from diseased raspberry plants.

The agar disk-diffusion method was used for the bioassay [72]. Small agar blocks (5 × 5 mm) with the mycelium of the corresponding isolate were cultured in the center of 90 mm Petri dishes. We grew the oomycete *Phytophthora cryptogea* on V8 Agar (16 g agar, 100 mL V8 Juice, and 900 mL distilled water) and the fungi *Botrytis cinerea* and *Fusarium oxysporum* on PDA (BD Difco™, BD, Franklin Lakes, NJ, USA). The Petri dishes were incubated overnight for a synchronized onset of growth before the application of the essential oils and acetone exudates. Four variants were performed with all the isolates: *A. santonicum* EO, *A. santonicum* AE, *A. lerchiana* EO, and *A. lerchiana* AE. The acetone exudates of each plant species were dissolved in DMSO at a concentration of 100 mg/mL, and two drops with a volume of 15 µL were dripped at equal distances from its center. In the same way, the essential oils were applied with a volume of 2 µL in pure form without dilution. Two control variants—one without treatment and one with DMSO—were also included in the experiment. Four replicates were made for each variant. The Petri dishes were cultivated in a climatic chamber at 25 °C in darkness. The results were documented after 6 days when fungi from the control variants of the three species reached the periphery of the Petri dishes. Photographs (Canon EOS 4000D, Canon, Tokyo, Japan) of all mycelial colonies were taken, and their mycelial growth areas were measured using the image analysis program ImageJ 1.54d [73]. Based on the obtained data (average mycelial growth area for each variant), the inhibition percentage was calculated using the following equation [21]:

IMG = 100 (C − T) C^−1^,

where IMG is the percentage of inhibition of mycelial growth, C is the area of the fungal colony without treatment (control), and T is the area of the fungal colony with treatment. Four replicates were performed for each variant.

### 4.9. Data Analysis

Statistical analyses were performed using Microsoft Excel software 2016. The AChE inhibitory data were analyzed using the software package Prism 3 (Graph Pad Inc., San Diego, USA). IC_50_ values were calculated using the same software.

## 5. Conclusions

The present study represents preliminary research on the pesticidal properties of *A. lerchiana* and *A. santonicum* essential oils and exudates. Both species are clearly distinguished by their essential oil profiles. The strong inhibitory activity on *L. perenne* seed germination and acetylcholinesterase was found by studying essential oils. However, essential oils showed weak inhibitory activity against the examined phytopathogens in the studied doses. The exudates of both species displayed phytotoxicity against *L. perenne* seed germination and weak AChE inhibitory activity. *A. lerchaiana* AE exhibited strong inhibitory activity against the mycelium growth of *Botrytis cinerea*. Essential oils and exudates of *A. lerchiana* and *A. santonicum* showed a phytotoxic potential that provides reasons for more detailed studies on their herbicidal activity. *Artemisia santonicum* EO is recommended to be examined for insecticidal activity and *A. lerchiana* AE for more extended studies on its inhibitory activity against *B. cinerea* and other pathogens. According to our knowledge, this is the first report of inhibitory activity on seed germination and acetylcholinesterase of *A. lerchiana* and *A. santonicum* essential oils and exudates.

## Figures and Tables

**Table 1:** Essential oil composition [area %] from the aerial parts of the studied *Artemisia* species.

RRI *	Compound	*A. lerchiana*	*A. santonicum*
914	2-Methyl-2,5-divinyltetrahydrofuran	0.8	-
929	α-Thujene	0.5	-
937	α-Pinene	0.3	9.3
952	Camphene	4.8	-
979	β-Pinene	0.3	15.2
991	2,3-Dehydro-1,8-cineole	0.3	-
1017	α-Terpinene	-	0.1
1024	*p*-Cymene-	1.2	5.3
1028	Limonene	-	2.7
1032	Eucalyptol (1,8-Cineole)	22.1	-
1052	*cis*-Arbusculone	0.1	-
1060	γ-Terpinene	0.5	0.4
1065	Acetophenone	-	0.2
1070	*trans*-Arbusculone	0.1	-
1070	*cis*-Sabinene hydrate	0.6	-
1088	Terpinolene	0.4	0.9
1090	*p*-Cymenene	0.1	0.1
1098	*trans*-Sabinene hydrate	0.2	-
1125	α-Campholenal	-	0.8
1139	*trans*-Pinocarveol	-	9.6
1140	*trans*-*p*-2-Menthen-1-ol	0.1	-
1142	*cis*-Verbenol	-	0.1
1145	Camphor	14.0	-
1157	Isoborneol	0.1	2.1
1163	Pinocarvone	-	3.0
1165	Borneol	6.6	-
1178	Terpinen-4-ol	2.1	0.1
1189	α-Terpineol	1.3	9.2
1195	Myrtenol	1.0	0.1
1205	Verbenone	-	0.1
1208	*cis*-Piperitol	1.4	-
1217	*trans*-Carveol	1.4	0.6
1233	*cis*-Carveol	0.1	-
1242	Carvone	0.6	0.8
1254	Piperitone	0.6	-
1258	Carvenone	0.6	-
1283	Lavandulyl acetate	0.1	-
1285	Bornyl acetate	1.1	2.7
1297	*trans*-Pinocarvyl acetate	-	0.1
1292	Thymol	0.2	0.1
1302	Carvacrol	6.1	3.8
1306	Isoascaridole	1.5	-
1327	Myrtenyl acetate	-	0.2
1347	Silphinene	-	0.1
1350	α-Terpinyl acetate	0.1	-
1354	Citronellol acetate	-	0.3
1357	Eugenol	0.3	-
1364	Neryl acetate	-	0.2
1379	Silphiperfol-6-ene	-	0.1
1382	Geranyl acetate	-	0.8
1382	Sabinyl propionate	-	0.9
1392	(*E*)-Jasmone	0.5	-
1402	Methyleugenol	0.1	0.1
1412	*cis*, *threo*-Davanafuran	0.2	-
1414	Sabinyl isobutanoate	-	0.2
1483	α-Curcumene	-	1.0
1485	β-Selinene	0.5	-
1490	Davana ether	1.0	-
1501	Capillene	-	4.2
1505	C_15_H_22_O (MW 220)	-	0.1
1510	Cameroonan-7α-ol	-	0.8
1515	Davana ether isomer	4.3	-
1530	Artedouglasia oxide C	1.8	-
1535	Artedouglasia oxide A	3.3	-
1544	Italicene ether	-	0.3
1566	Artedouglasia oxide D	1.1	-
1573	1,5-Epoxysalvial-4(14)-ene	-	1.4
1578	Artedouglasia oxide B	1.8	-
1580	Spathulenol	1.8	8.5
1595	Viridiflorol	0.9	-
1608	β-Antlantol	-	2.1
1639	Capillin	-	3.2
1655	C_15_H_24_O (MW 220)	2.8	-
1660	Neointermedeol	3.3	-
1677	Valeranone	-	0.7
1688	Eudesma-4(15),7-dien-1β-ol	-	1.1
1692	C_15_H_26_O (MW 222)	1.0	-
1752	γ-Costol	0.2	-
1776	α-Costol	0.4	-
1820	(*E*)-Artemidin	-	3.1
1835	C_15_H_20_O_3_ (MW 248)	0.4	-
1844	Hexahydrofarnesyl acetone	0.1	-
1880	(*Z*)-Artemidin	-	1.5
	Monoterpene hydrocarbons	8.2	34.0
	Oxygenated monoterpenes	62.7	35.9
	Sesquiterpene hydrocarbons	0.5	1.2
	Oxygenated sesquiterpenes	24.4	19.5
	Other	1.3	7.6
	Total	97.1	98.1

* Relative retention index (RRI) determined relative to a homologous series of *n*-alkanes (C_8_–C_25_) on the HP-5MS column.

**Table 2:** Exudate composition [area %] from the aerial parts of the studied *Artemisia* species.

RRI *	Compound	*A. lerchiana*	*A. santonicum*
1022	Eucalyptol	6.45	-
1070	Glycolic acid	0.34	-
1150	Camphor	4.58	-
1161	Borneol	3.69	-
1177	Terpinen-4-ol	0.2	-
1195	Myrtenol	1.95	-
1253	Succinic acid	0.1	0.39
1255	Ascaridole	1.49	-
1260	Glycerol	0.86	2.1
1265	Octanoic acid	-	2.27
1289	Bornyl acetate	0.37	-
1294	4-Methoxyphenol	-	0.95
1337	Carvacrol	1.86	0.18
1340	Glyceric acid	-	0.45
1345	Fumaric acid	-	4.25
1393	Hydroquinone	-	1.9
1436	*trans*-Cinnamic acid	-	0.95
1461	4′-Hydroxyacetophenone	0.21	0.42
1480	10-Undecenoic acid	11.45	-
1481	β-Selinene	0.63	-
1498	Malic acid	-	5.72
1500	Capillene	-	1.32
1503	*meso*-Erythritol	-	0.1
1515	Pyroglutamic acid	-	1.63
1536	Artedouglasia oxide A	0.45	-
1562	Methyl *p*-coumarate	0.19	-
1578	Erythronic acid	-	2.45
1640	4-Hydroxybenzoic acid	0.2	0.1
1660	Neointermedeol	1.23	-
1700	Methyl 3,4-dihydroxybenzoate	-	0.31
1774	Costol	0.76	-
1776	Vanillic acid	0.2	0.25
1790	1,2-Longidione	-	0.83
1811	Protocatechuic acid	0.33	0.3
1813	Pinitol	-	3.61
1816	2-Ethylhexyl salicylate	0.94	-
1845	Fructose	0.21	0.41
1849	Quinic acid	0.51	0.96
1880	Syringic acid	0.1	-
1940	Hydroxycinnamic acid	0.1	0.1
1972	Glucose	0.15	0.21
2042	Hexadecanoic acid (*palmitic acid*)	0.34	0.47
2097	Ferulic acid	0.21	
2126	Myo-Inositol	0.31	0.28
2137	Caffeic acid	-	0.3
2162	Octadecanoic acid (*stearic acid*)	-	0.14
2201	9,12-Octadecadienoic acid (*linoleic acid)*	-	0.22
2568	rac-Glycerol 1-palmitate	-	0.34
2709	Sucrose	0.80	1.45
2757	Glycerol monostearate	-	0.20
3100	Chlorogenic acid	-	0.68
3149	6-O-Methylapigenin (*hispidulin*)	0.1	-
3198	6-O-Hydroxyluteolin 4′,6-dimethl ether (*jaceosidin*)	1.05	-
3325	β-Amyrin	0.1	0.2
3355	α-Amyrin	0.87	1.27
3402	6-O-Hydroxyluteolin 4′,6,7-trimethyl ether (*eupatorin*)	0.94	-

* Relative retention index (RRI) determined relative to a homologous series of *n*-alkanes (C_8_–C_25_) on the HP-5MS column.

**Table 3:** Acetylcholinesterase inhibitory activity of *A. lerchiana* and *A. santonicum* EO and AE.

Studied Fraction	IC_50_, [μg/mL]	
	*A. lerchiana*	*A. santonicum*
Essential oils	64.42 ± 2.69	14.60 ± 0.58
Acetone exudates	961 ± 0.17	>1000
Galanthamine (positive control)	0.21 ± 0.02	

**Table 4:** Used solutions and concentrations of EO and AE in the studied bioassays.

Bioassay	Studied Fraction	Solvent	Tested Dose/Concentration
Inhibition of seed germination	EO	0.1% solution Tween 40	0.5 to 3.0 μL/mL
	AE	water:acetone 99.5:0.5 (*v*/*v*)	1 to 8 mg/mL
Inhibition of acetylcholinesterase	EO	Methanol	0.001 to 1000 µg/mL
	AE	Methanol	0.001 to 1000 µg/mL
Inhibition of phytopathogenic mycelium growth	EO	Without	0.2 µL
	AE	DMSO	15 µL (100 mg/mL)

## Data Availability

Not applicable.
